# Analysis of Hazy Ga- and Zr-Co-Doped Zinc Oxide Films Prepared with Atmospheric Pressure Plasma Jet Systems

**DOI:** 10.3390/nano13192691

**Published:** 2023-10-01

**Authors:** Yu-Tang Luo, Zhehan Zhou, Cheng-Yang Wu, Li-Ching Chiu, Jia-Yang Juang

**Affiliations:** 1 Department of Mechanical Engineering, National Taiwan University, Taipei 10617, Taiwan; r09522552@ntu.edu.tw (Y.-T.L.); r10522549@ntu.edu.tw (Z.Z.); r09522520@ntu.edu.tw (C.-Y.W.); r09522531@ntu.edu.tw (L.-C.C.); 2 Program in Nanoengineering and Nanoscience, Graduate School of Advanced Technology, National Taiwan University, Taipei 10617, Taiwan

**Keywords:** transparent conductive oxide (TCO), atmospheric pressure plasma jet (APPJ), co-doping, haze

## Abstract

Co-doped ZnO thin films have attracted much attention in the field of transparent conductive oxides (TCOs) in solar cells, displays, and other transparent electronics. Unlike conventional single-doped ZnO, co-doped ZnO utilizes two different dopant elements, offering enhanced electrical properties and more controllable optical properties, including transmittance and haze; however, most previous studies focused on the electrical properties, with less attention paid to obtaining high haze using co-doping. Here, we prepare high-haze Ga- and Zr-co-doped ZnO (GZO:Zr or ZGZO) using atmospheric pressure plasma jet (APPJ) systems. We conduct a detailed analysis to examine the interplay between Zr concentrations and film properties. UV-Vis spectroscopy shows a remarkable haze factor increase of 7.19% to 34.8% (+384%) for the films prepared with 2 at% Zr and 8 at% Ga precursor concentrations. EDS analysis reveals Zr accumulation on larger and smaller particles, while SIMS links particle abundance to impurity uptake and altered electrical properties. XPS identifies Zr mainly as ZrO_2_ because of lattice stress from Zr doping, forming clusters at lattice boundaries and corroborating the SEM findings. Our work presents a new way to fabricate Ga- and Zr-co-doped ZnO for applications that require low electrical resistivity, high visible transparency, and high haze.

## 1. Introduction

Transparent conductive oxides (TCOs) have garnered significant attention for their unique combination of low electrical resistivity and high visible light transparency, rendering them indispensable in various optoelectronic and photovoltaic devices. Notable TCOs such as indium tin oxide (ITO) [1,2,3], fluorine-doped tin oxide (FTO) [4,5,6], aluminum-doped zinc oxide (AZO) [7,8], and gallium-doped zinc oxide (GZO) [9,10,11] have been extensively explored for applications spanning electrochromic glasses [12,13], touch screens [14], flat panel displays [15], and thin-film solar cells [16,17,18]. While ITO has dominated the field, its impending scarcity and rising cost due to limited indium reserves have motivated the pursuit of alternatives. ZnO-based TCOs, with their favorable attributes, including non-toxicity, cost-effectiveness, ease of preparation, and robust optoelectronic properties, hold promise as suitable alternatives [19,20].

ZnO, exhibiting a hexagonal wurtzite-type crystalline structure, features a wide direct band gap of 3.37 eV. Through the strategic incorporation of extrinsic dopants such as aluminum (Al) and gallium (Ga), its resistivity can be markedly reduced to a range of 2–4 × 10^−4^ Ω cm. Extensive research has also demonstrated the efficacy of doping with diverse elements spanning groups IIIA (B, In), IIIB (Sc, Y, La), IVA (Ti, Zr, Hf), IVB (Si, Ge, Sn), and non-metal anions (Cl, F). Beyond this, the concept of “co-doping” or “double doping” ZnO has been investigated to enhance conductivity further, offering avenues for superior performance [21]. This wealth of research on co-doping focuses on the electrical attributes of thin films, with limited attention directed toward their optical characteristics, particularly haze properties. “Haze” (*H*) is an optical parameter used to discuss the light-scattering ability of a thin film. Thin films with light-scattering abilities have advantages in increasing the optical path length in photovoltaic applications. The definition of this parameter is as follows:

(1) $$H(\lambda )=\frac{T_{t}(\lambda )-T_{s}(\lambda )}{T_{t}(\lambda )}=\frac{T_{d}(\lambda )}{T_{t}(\lambda )}$$

where *T_t_*, *T_s_*, and *T_d_* are the total, specular, and diffuse transmittances, respectively; *λ* is the wavelength.

Our previous work introduced a novel Ga- and Zr-co-doped ZnO formulation (GZO:Zr or ZGZO) that exhibits remarkable and tunable haze while concurrently maintaining favorable resistivity and optical transmittance [22]. Integrating textured and hazy front electrodes into solar cell applications has been shown to augment light-scattering effects, enhancing photon absorption and overall power conversion efficiency. However, it is imperative to note that high haze can influence the film’s resistivity and optical transmittance, presenting a complex trade-off [23]. Consequently, a thorough and application-specific analysis is warranted.

Conventionally, the fabrication of textured or hazy TCOs necessitates additional steps or supplementary layers. Examples include substrate etching pre-deposition or film surface etching post-deposition [24,25] and incorporating haze-enhancing layers or nanowires/nanoparticles into substrates [26]. These techniques, however, augment processing complexity and associated costs. By contrast, the innovative approach of depositing hazy GZO:Zr thin films using the same apparatus and methodology employed for single-dopant ZnO deposition allows for a controllable haze of up to ~46% [22].

In the present study, we delve into a comprehensive investigation of the mechanism underlying the formation of spherical particles on textured GZO:Zr films, encompassing detailed assessments of chemical composition through the application of energy-dispersive X-ray spectroscopy (EDS), secondary ion mass spectrometry (SIMS), and X-ray photoelectron spectroscopy (XPS). This comprehensive approach offers valuable insights into the intricacies of these textured thin films, providing a foundation for optimizing their optoelectronic characteristics in the context of solar cell applications.

## 2. Experimental Details

### 2.1. Film Deposition

**Figure 1:**
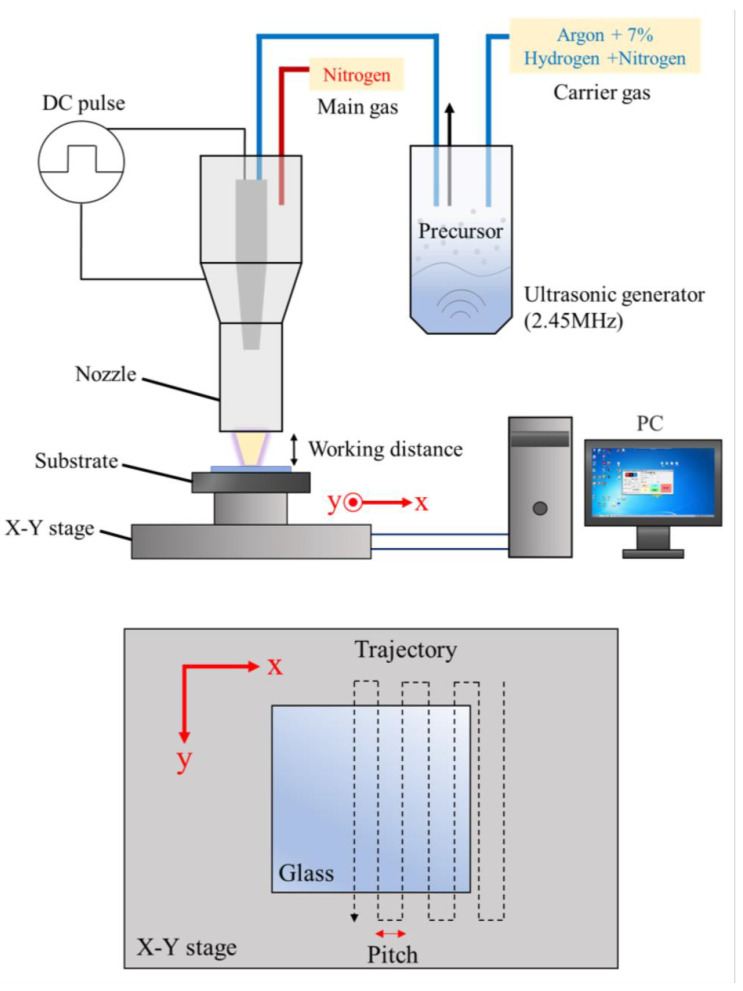
The schematics of the APPJ system (top panel) and nozzle scanning trajectory (bottom panel). In one spray, the X-Y stage moves backward and forward in the *y*-direction at a constant speed of 10 mm/s and moves at a pitch of 2 mm in the *x*-direction, depicted as the dotted line. The red arrows are the coordinate axes.

GZO:Zr thin films were deposited on glass substrates (Asahi Glass Co. Ltd., Tokyo, Japan, RLO-0.5, soda-lime alkali glass, 50 mm × 50 mm × 0.5 mm dimensions) under ambient conditions using an atmospheric pressure plasma jet (APPJ) system. The APPJ system, illustrated in Figure 1, consists of a plasma jet, a direct-current (DC) pulse power supply, an ultrasonic generator, a heating plate, and a computer-controlled X-Y platform. The main gas of the plasma is N_2,_ and the flow rate is set to 20 slm. The plasma is generated by a 160-V DC pulse power supply with a repetition rate of 27.78 kHz and a duty cycle of 22.2% (the on/off duration of the power supply is 8/28 μs); the transformer ratio is 1:40, which can amplify the voltage to 6.4 kV. We used 20 slm of nitrogen as the main gas and a mixture of N_2_ (140 sccm) and forming gas (60 sccm, 93% Ar + 7% H_2_) as the carrier gas. More technical details about the APPJ system can be found in our previous work [22,26,27,28,29]. The substrate temperature was maintained at 180 °C. For the uniformity of the film, we set the working distance (the distance between the set nozzle and the glass) as 1.75 mm, the pitch in the x direction as 2 mm, and the nozzle would scan through the entire substrate twice at a speed of 10 mm/s.

To deposit GZO:Zr films, we used zinc nitrate (Zn(NO_3_)_2_, J.T. Baker, 99.6% purity), gallium nitrate (Ga(NO_3_)_3_, Alfa Aesar, 99.9% purity), and zirconyl nitrate (ZrO(NO_3_)_2_, Aldrich, 99% purity) dissolved in ionized water as precursors; the concentration of the solution was kept at 0.6 M, and the atomic percentage of Ga/(Ga + Zn + Zr) was set as 8%. The value of Zr/(Ga + Zn + Zr) varied from 0% to 6%, and for simplicity, we express the samples as 0 at%, 2 at%, 4 at%, and 6 at% (for example, a “2 at% sample” has 2 at% Zr, 8 at% Ga, and 90 at% Zn). Note that these are the concentrations in the precursor and may not be the same as the doping concentration in the deposited films. It is, however, expected that the doping concentration will be positively correlated with the precursor concentration.

### 2.2. Film Characterization

Scanning electron microscopy (SEM, JSM-7800F, Akishima, Tokyo, Japan) was performed to observe the microstructures of the thin films from the top view. The optical properties were measured with a UV-Vis-NIR spectrometer (Jasco V-670, Easton, MD, USA). Energy-dispersive X-ray spectroscopy (EDS, JSM-7800F Prime, Akishima, Tokyo, Japan) was performed to analyze the elemental composition ratio of the thin film. Secondary ion mass spectrometry (TOF-SIMS IV, Munster, Germany) was employed to characterize the element distribution of subsurface regions. X-ray photoelectron spectroscopy (XPS) was used to analyze the chemical composition of the films.

## 3. Results

### 3.1. Morphological Properties

**Figure 2:**
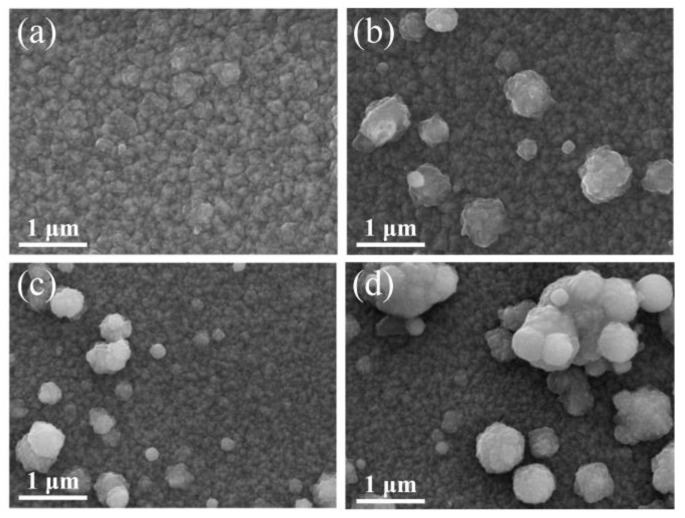
SEM image of different Zr concentrations: (**a**) GZO (0 at%); (**b**) GZO:Zr (2 at%); (**c**) GZO:Zr (4 at%); (**d**) GZO:Zr (6 at%).

Figure 2 exhibits the effect of different Zr concentrations on the surface morphology of the GZO and GZO:Zr thin films. The images show that spherical particles appear on the surface of the co-doped GZO:Zr films and the number of these spherical particles increases with the Zr concentration. Given the increase in the number of particles, the coverage of the particles also increases with the Zr concentration.

It has been reported that by using the APPJ system and the same precursor ZrO(NO_3_)_2_·xH_2_O as in this work, tetragonal ZrO_2_ particles can be prepared [30]. The study proposes a reaction formula as follows.

(2) $${\mathrm{ZrO}(\mathrm{NO}}_{3}{)}_{2}\to {\mathrm{ZrO}}_{2}+2{\mathrm{NO}}_{2}↑+\frac{1}{2}O_{2}↑$$

It can be seen from the formula that ZrO(NO_3_)_2_ turns into ZrO_2_ after a plasma-assisted reaction. Therefore, the resultant film may also contain ZrO_2_ in addition to the intended GZO:Zr.

The phenomenon of spherical particles appearing on the surface of the film because of co-doping has also been mentioned in previous studies; it was reported that spherical particles were found on the surface of Al-B-co-doped ZnO and Al-Ga-co-doped ZnO [31]. That research inferred that the different growth rates of different crystallographic directions of the film may cause the production of these spherical particles. Note that the presence of ZrO_2_ degrades the electrical properties of the deposited films [22]. We did not investigate possible processes to minimize its presence in the present work. However, our findings show that ZrO_2_ on a film surface considerably increases the haze of the film, which may be beneficial for some applications [22]. Thus, the Zr concentration should be carefully selected according to the desirable haze and optoelectronic properties.

### 3.2. Optical Properties

**Figure 3:**
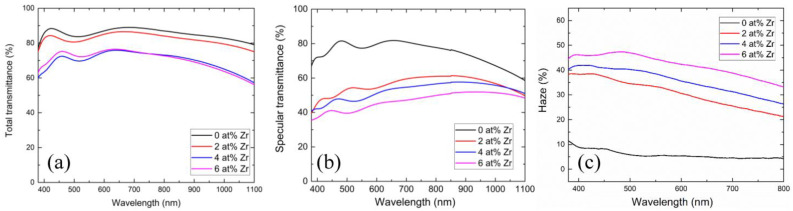
(**a**) Total transmittance of samples with different Zr concentrations. (**b**) Specular transmittance of samples with different Zr concentrations. (**c**) Haze of samples with different Zr concentrations.

Figure 3a shows the total transmittance of samples with different Zr concentrations. As illustrated in the figure, the total transmittance of the film experiences a decline as the Zr concentration increases. This phenomenon is attributed to the fact that absorption is closely related to thickness—absorption diminished exponentially as the film thickness decreased. In our previous work, the thickness of each sample was detailed, revealing that our film’s thickness varies with alterations in Zr concentration. Specifically, the sample GZO (0 at%), GZO:Zr (2 at%), GZO:Zr (4 at%), and GZO:Zr (6 at%) exhibited thicknesses of approximately 531 nm, 658 nm, 656 nm, and 768 nm, respectively. For details regarding the measurements of the GZO:Zr film thicknesses at these four Zr concentration levels, we refer the reader to our prior work [22]. Notably, within the visible spectrum (380 nm–800 nm), the total transmittance of GZO:Zr (2 at%) approaches 82%, and a similar high transmittance of nearly 80% is also observed in the near-infrared region (800 nm–1100 nm), which includes a ~10% loss due to the glass substrate.

Specular transmittance (*T_s_*) constitutes a portion of the total transmittance (*T_t_*), where light traverses the film and substrate without undergoing scattering. In Figure 3b, the specular transmittance of samples with different Zr concentrations is displayed. Similar to the total transmittance, the specular transmittance of the film decreases with increasing Zr concentrations. Moreover, it is noteworthy that the drop in specular transmittance is more pronounced than in total transmittance. This phenomenon can be attributed to the presence of spherical particles, which contribute to surface roughness and subsequently lead to light scattering when traversing both the film and substrate. Consequently, the reduction in specular transmittance exceeds the reduction in total transmittance [32,33].

Figure 3c illustrates the haze characteristics of samples at varying Zr concentrations. Given that a reduction in specular transmittance corresponds to an increase in haze, it follows that, as the Zr concentration rises, the haze also increases. This finding further corroborates the role of spherical particles in augmenting the film’s surface roughness, resulting in an increase in haze. As an example, for GZO prepared with 2at% Zr, the haze increases substantially from 7.19% to 34.8% at a wavelength of 550 nm, representing a remarkable increment of +384%.

TCO layers are typically around 100 nm in thickness. However, it is important to note that our GZO:Zr films exhibited relatively high resistivity compared with ITO or FTO, necessitating a greater thickness to achieve a desirable sheet resistance. In our prior investigation [22], we reported that GZO:Zr films prepared with a 2% Zr concentration demonstrated an optical transmittance of approximately 80% and a sheet resistance of 13 Ω/sq. Furthermore, we conducted a comparison of the Figure of Merit (FoM) with results from the existing literature, revealing that our FoM is either comparable to or lower than that of most TCO materials.

### 3.3. EDS Analysis

**Figure 4:**
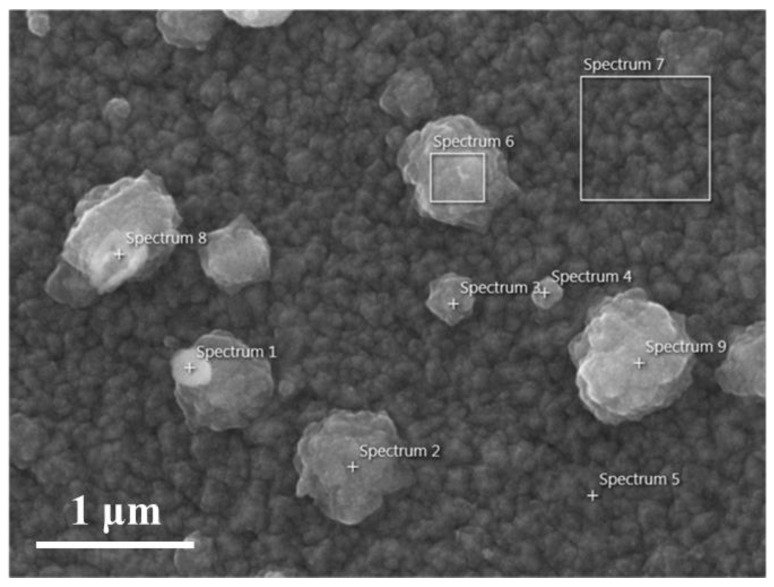
SEM image of GZO:Zr (2 at%) with 9 spectrums.

We selected nine regions on the SEM image of GZO:Zr (2 at%) (as shown in Figure 4) and performed EDS analysis to determine the chemical composition of the spherical particles on the film surface. We summarized the analyzed results in Table 1. In Table 1, the carbon content of the spheres is more than that of the film surface. We suggest that this is because these particles’ existence increases the film’s surface area. The larger surface area increases the likelihood of adsorbing contaminants in the air, resulting in higher carbon content in the film. We found that the Zr tended to gather on the edge of the larger spherical particle and all of the smaller spherical particles, and there were few Zr particles in the center of the larger spherical particle and the surface of the film.

To explain the phenomenon observed here, we recall the XRD result reported in our previous work [22], in that the crystallization size decreases as the Zr concentration increases. It is known that the ionic radius difference between Zn and Zr can cause a large internal stress inside the ZnO lattice [34,35], which might result in high structural stains and reduce the crystallinity of the GZO:Zr film. Since Zr ions may distort the crystal structure of the ZnO lattice, they tend to accumulate on the particle edge or grain boundaries.

### 3.4. SIMS Analysis

**Figure 5:**
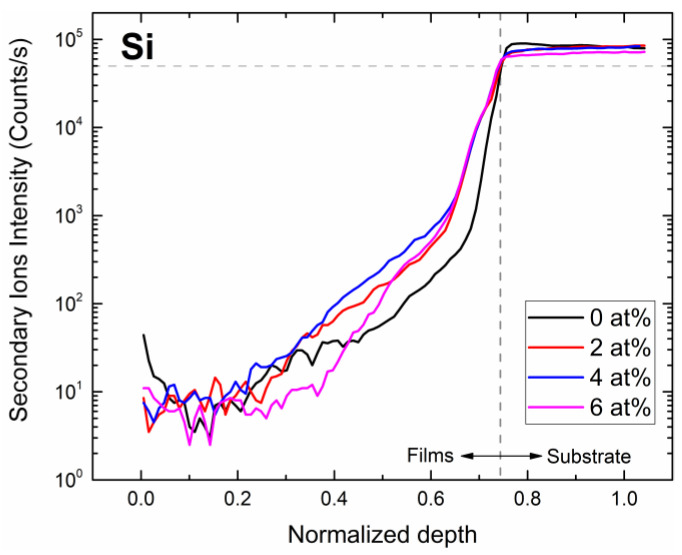
Si SIMS measurement of samples with different Zr concentrations.

**Figure 6:**
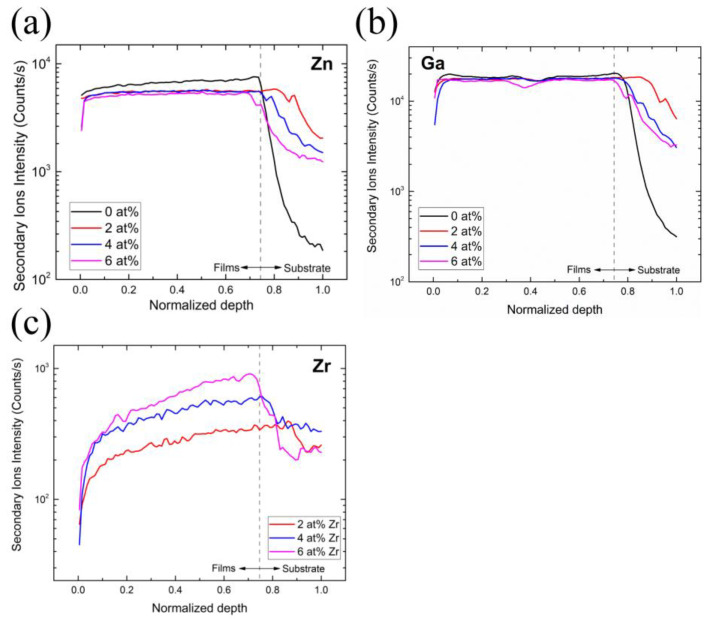
SIMS measurement of samples with different Zr concentrations: (**a**) Zn, (**b**) Ga, (**c**) Zr.

The elemental distribution of the subsurface region for silicon (Si), zinc (Zn), gallium (Ga), and zirconium (Zr) elements for samples with different Zr concentrations (Figure 5 and Figure 6) were determined using SIMS depth profile analysis. Since Si is a primary element of the glass substrate and the deposited film contains almost no Si, it is helpful to locate the film–substrate interface using Si content. Since film thickness varies with the Zr concentration, we normalized the thickness to a range between 0 and 1 to facilitate the comparison. We can determine the depth of the interface between films and substrates through the investigation of Si. The depth of the interface is defined as the position where the ion intensity of Si is half of the maximum.

According to Figure 6a, it can be seen that, when the Zr concentration increases, the ion intensity of the Zn tends to decrease. This can be explained by the fact that, in all of our experiments, the precursor concentration of Ga was set to 8%, so when the Zr concentration increased, the Zn concentration decreased in the same proportion. This can also explain the largely invariant distributions of Ga ion intensity at different Zr concentrations, as shown in Figure 6b.

Lastly, the concentration profile of Zr (Figure 6c) shows that the ion intensity of the Zr increases with the Zr concentration. In addition, the tailing phenomenon can be observed in the element distribution curves of Zn, Ga, and Zr, and from the distribution curve of the Si, it can also be seen that the signal of Si is also detected in the film, which is caused by the element diffusion phenomenon.

### 3.5. XPS Analysis

In this work, XPS analysis was performed to understand the mechanism of co-doping on thin film, focusing on the binding energy of three elements: Ga2p, O1s, and Zr3d with different Zr concentrations.

**Figure 7:**
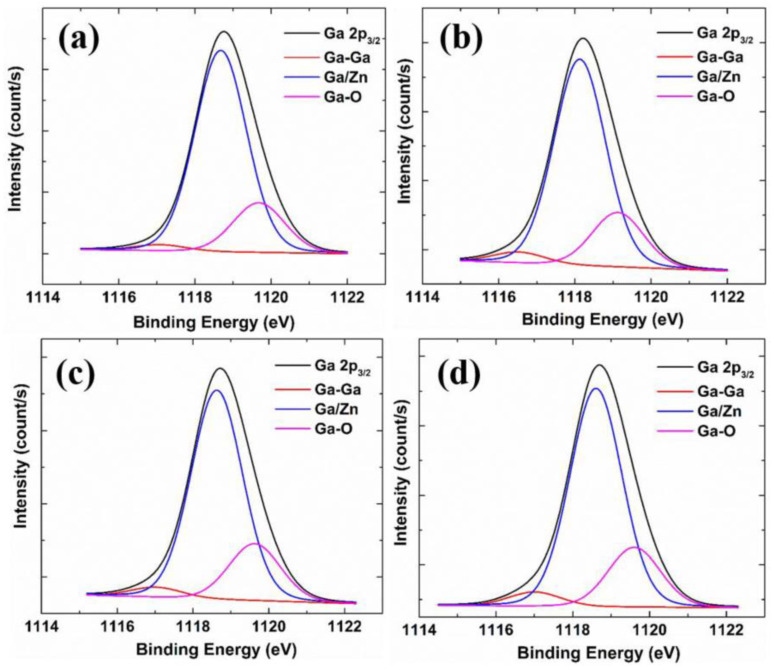
XPS spectra of Ga 2p_3/2_ states of different samples: (**a**) GZO; (**b**) GZO:Zr; (2 at%); (**c**) GZO:Zr (4 at%); (**d**) GZO:Zr (6 at%).

Figure 7a compares the binding energy characteristics of the Ga element in films with different doping concentrations. The Ga 2p_3/2_ orbital domain can be divided into three binding types, which are the metallic-gallium-binding energy at 1116.8 eV (expressed as Ga-Ga) and the binding energy of Ga^3+^ replacing Zn^2+^ in the lattice at 1118.4 eV (expressed as Ga/Zn). Moreover, the gallium–oxygen-binding energy is 1119.4 eV (expressed as Ga-O). The Ga-O bond may come from Ga_2_O_3_ or GaO_x_ clusters. When the carrier concentration is too high (N ≧ 5 × 10^20^ cm^−3^), part of the dopant may not be activated. Instead, they tend to aggregate into clusters in grains or grain boundaries, decreasing carrier concentration and Hall mobility. Table 2 lists the ratios of the three bonding types of four films with different Zr concentrations. We find that there are appreciable differences between GZO and co-doped GZO:Zr samples in the ratios of Ga/Zn and Ga-O. However, these ratios remain largely unchanged as the Zr concentration increases. This may be attributed to the fact that the Ga atomic ratio of the GZO:Zr precursors is fixed. Recall that Ga/(Ga + Zn + Zr) was set as 8%. This phenomenon can also be observed in the SIMS measurement in Figure 6b.

**Figure 8:**
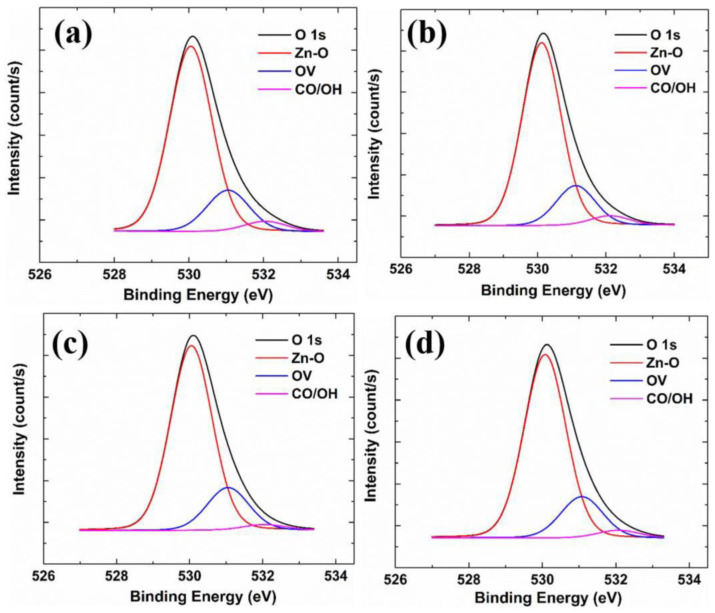
XPS spectra of the O 1s state of different samples: (**a**) GZO; (**b**) GZO:Zr (2 at%); (**c**) GZO:Zr (4 at%); (**d**) GZO:Zr (6 at%).

Figure 8 shows the XPS spectra of the O 1s state of samples with different Zr concentrations. The O 1s orbital domain can be divided into three bonding types: (i) Covalent oxygen (expressed as Zn-O) at 530.15 eV, which is the oxygen bonded to Zn ions. (ii) Oxygen vacancies at 531.25 eV (expressed as OV), which is the defect formed by the oxygen in the lattice, leaving the lattice because of external factors; oxygen vacancies can provide free carriers in n-type semiconductors, increasing the carrier concentration. (iii) Chemisorbed oxygen at 532.40 eV (expressed as CO/OH), which is related to impurities in the atmosphere, oxygen and water molecules in the atmosphere, and carbonate ions (CO_3_^−^) from the glass substrate. In a high-temperature deposition environment, chemisorbed oxygen tends to accumulate on the grain boundaries to form carrier traps, decreasing carrier concentration and hindering carrier mobility. In Table 3, it can be seen that the proportions of the three bonds have no apparent trend.

**Figure 9:**
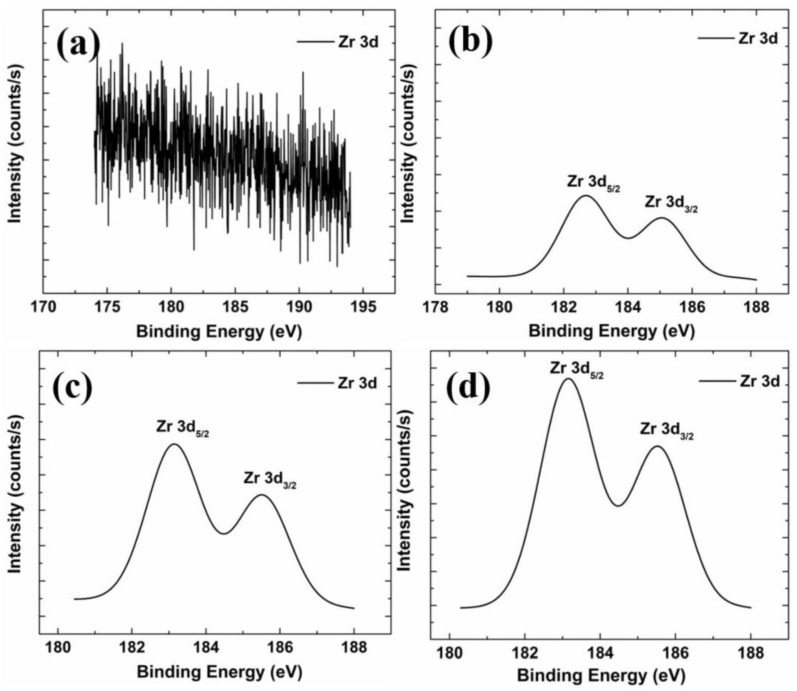
XPS spectra of the Zr 3d state of different samples: (**a**) GZO; (**b**) GZO:Zr (2 at%); (**c**) GZO:Zr (4 at%); (**d**) GZO:Zr (6 at%).

Figure 9 shows the binding energy of Zr 3d elements in films with different Zr concentrations. The Zr 3d data of the non-doped Zr film is mostly noise, which suggests that the film does not contain Zr. As the Zr concentration increases, the Zr 3d signal increases. The area under the Zr 3d peak is listed in Table 4. The Zr content ratio of each thin film is about X:1:1.9:2.9, which is quite close to the X:1:2:3 ratio of Zr content in the precursor. Zr 3d can be divided into two peaks, Zr 3d_5/2_ and Zr 3d_3/2,_ because of spin–orbit splitting. Table 4 lists the peak positions of Zr 3d_5/2_ and Zr 3d_3/2_ in each film. The two peaks fall at about 183.0 eV, and the position of 185.0 eV is known from the literature. When Zr 3d_5/2_ and Zr 3d_3/2_ are located at 182.6 eV and 185.0 eV, respectively, it means that Zr exists in the Zr-O bond in the form of Zr^4+^, but it cannot be determined that it is a substitution lattice. The Zr-O bond formed by Zn in the film or the Zr-O bond formed by the cluster of ZrO_2_ must be judged in conjunction with the trend of electrical properties [36]. The specific trend of electrical properties can be compared with the film thickness. In our previous work, we suggest that 2 at% Zr is enough to reach the film’s solubility limit, so most of the Zr-O bonds may appear in the film as ZrO_2_ clusters. Only a small part of Zr replaces Zn in the lattice, producing carriers. In addition, the study by Lackner et al. also used XPS to analyze the chemical composition of tetragonal ZrO_2_ [37]. Their results showed that the peak positions of Zr 3d_5/2_ and Zr 3d_3/2_ were located at 182.9 eV and 185.4 eV, respectively, consistent with the present study. Thus, it may be inferred that most of the Zr in the thin film is in the form of ZrO_2_.

With the discussion above, we infer that most Zr ions in the film did not replace Zn in the lattice site but instead formed ZrO_2_ clusters. Our previous work mentioned that the additional Zr ions in the ZnO lattice site would cause extra structural strain. Therefore, it is reasonable to assume that most of the Zr ions in the ionized precursor are kept out of the ZnO lattice and form ZrO_2_ independently. Moreover, with the SIMS and EDS analysis discussed above, we can find that these ZrO_2_ clusters are pushed on the film’s surface, forming the characteristic spherical particle morphology of GZO:Zr film.

## 4. Conclusions

In this study, we analyzed the morphological, optoelectronic, and chemical compositions of Ga- and Zr-co-doped ZnO films (GZO:Zr or ZGZO) synthesized using APPJ systems. Our thorough examination using SEM revealed the distinctive emergence of spherical particles on the film surface, a direct outcome of Zr doping. By employing UV-Vis spectroscopy, we unveiled a significant increase in the haze factor, soaring from 7.19% to a remarkable 34.8% (+384%), which we attributed to incorporating a 2 at% Zr precursor concentration. Our EDS measurements delineated a pronounced Zr accumulation at the periphery of larger spherical particles and their presence across all smaller ones. We carried out SIMS analysis on Si, Zn, Ga, and Zr for samples with different Zr concentrations. We found that Ga content was mainly invariant, whereas Zn content slightly decreased as the Zr content increased in our experimental conditions. We used Si distribution to locate the film—substrate interface and to determine the film thickness with high accuracy.

We employed XPS to elucidate that the Zr species predominantly manifest in the film as ZrO_2_. This phenomenon stems from the inherent increase in internal stress within the ZnO lattice consequent to Zr doping. This elevation in internal stress fosters the autonomous aggregation of Zr elements, culminating in the formation of ZrO_2_ clusters. Notably, these clusters congregate at the boundaries of the ZnO lattice, in agreement with our EDS observations, effectively elucidating the origin of the observed spherical particles on the film surface.

In summary, our methodology for fabricating high-quality hazy films eliminates the need for a vacuum environment or additional steps, thus streamlining the processing approach. Our findings may shed light on the study of co-doped ZnO with controllable haze.

## Figures and Tables

**Table 1:** Comparison of the chemical composition of the 9 spectrums of GZO:Zr (2 at%).

	S1	S2	S3	S4	S5	S6	S7	S8	S9
O (at%)	50.4	45.2	47.8	47.2	42.4	45.6	45.6	46.1	45.4
Zn (at%)	17.3	24.9	23.5	21.4	31.6	23.7	27.7	18.6	20.8
Ga (at%)	0.9	1.4	1.2	1.1	1.4	1.3	1.4	1.2	1.4
Zr (at%)	3.6	0.4	1.7	1.3	N/A	0.5	0.3	1.5	0.3

**Table 2:** Comparison of XPS data for the Ga 2p_3/2_ core levels of different samples.

	Ga-Ga	Ga/Zn	Ga-O
GZO	2.4%	78.3%	19.3%
GZO:Zr (2 at%)	4.1%	75.9%	20.0%
GZO:Zr (4 at%)	3.7%	75.7%	20.6%
GZO:Zr (6 at%)	4.9%	74.7%	20.4%

**Table 3:** Comparison of XPS data for the O 1s core levels of different samples.

	Zn-O	OV	CO/OH
GZO	78.2%	17.4%	4.3%
GZO:Zr (2 at%)	78.9%	17.1%	4.0%
GZO:Zr (4 at%)	79.4%	18.3%	2.3%
GZO:Zr (6 at%)	78.9%	17.8%	3.3%

**Table 4:** Peak positions of Zr 3d_3/2_ and Zr 3d_5/2_ of different samples.

	Zr 3d_5/2_ (eV)	Zr 3d_3/2_ (eV)	Area under Zr 3d	Ratio
GZO	N/A	N/A	N/A	N/A
GZO:Zr (2 at%)	182.7	185.1	518.4	1
GZO:Zr (4 at%)	183.1	185.5	1000.1	1.9
GZO:Zr (6 at%)	183.1	185.5	1519.3	2.9

## Data Availability

The data presented in this study are available upon request from the corresponding author.
